# Neuropsychiatric and Neurocognitive Manifestations in HIV-Infected Children Treated With Efavirenz in South Africa—A Retrospective Case Series

**DOI:** 10.3389/fneur.2019.00742

**Published:** 2019-07-09

**Authors:** Charles K. Hammond, Brian Eley, Natalia Ing, Jo M. Wilmshurst

**Affiliations:** ^1^Department of Paediatric Neurology, Neuroscience Institute, Red Cross War Memorial Children's Hospital, University of Cape Town, Cape Town, South Africa; ^2^Department of Child Health, Kwame Nkrumah University of Science and Technology, Kumasi, Ghana; ^3^Paediatric Infectious Diseases Unit, Red Cross War Memorial Children's Hospital, University of Cape Town, Cape Town, South Africa; ^4^Department of Psychology, Paediatric Neuropsychology Clinic, University of Cape Town, Cape Town, South Africa

**Keywords:** efavirenz, neuropsychiatric, neurocognitive, adverse effect, neurotoxicity, children

## Abstract

**Background:** Efavirenz is associated with transient neuropsychiatric manifestations but the impact on neurocognition in children is unknown. Genetically slow metabolizers of efavirenz may be at risk of toxicity. This study describes neuropsychiatric and neurocognitive manifestations of South African children with suspected efavirenz neurotoxicity.

**Method:** This retrospective study describes clinical features of 12 children with features consistent with efavirenz neurotoxicity between 2008 and 2014.

**Results:** Twelve children (4 males, 8 females) aged 3–12 years (median 8.4 years) were referred to a dedicated pediatric neuroHIV service. Eight were of indigenous African (black) ancestry and 4 were of mixed ancestry. The total duration on efavirenz-containing ART at the time of referral was 6–72 (mean 31) months. Two children (both of black ancestry) were phenotypically slow metabolizers and presented with acute manifestations and high plasma efavirenz concentrations above normal range resulting in discontinuation of efavirenz. Ten other children had clinical presentations compatible with efavirenz neurotoxicity but had normal or sub-therapeutic plasma efavirenz concentrations and continued treatment with efavirenz. The acute neuropsychiatric manifestations reported included drowsiness, seizures, sleep disturbances, personality changes, ataxia, and slurred speech. These were noticed 2–8 weeks (mean 5 weeks) after commencing efavirenz and resolved over a few weeks. Nine children had neurocognitive deficits and showed poor performance in all neurocognitive domains that were tested.

**Interpretation:** Efavirenz causes transient neuropsychiatric adverse effects and may contribute to poor long-term neurocognitive outcomes in HIV-infected children. Prospective studies comparing efavirenz-treated and efavirenz-naïve children are needed to further elucidate the manifestations of efavirenz toxicity.

## Background

By 2016 an estimated 320,000 children with HIV infection, between 0 and 14 years of age, were living in South Africa, with antiretroviral therapy (ART) coverage reaching about 55% (1). Efavirenz (EFV) is a highly potent first-line ART drug widely used by HIV-infected children and adults. A once-daily dose is usually given at night. Despite its efficacy and ease of administration, there is emerging clinical evidence, supported by experimental data, that it causes damage to neurons with resultant neuropsychiatric adverse effects (2).

Transient neuropsychiatric effects occur in up to 50% of people receiving EFV and include dizziness, blurred vision, impaired concentration, or drowsiness, insomnia, nightmares, headache, mood changes and seizures (3–5). Psychiatric manifestations, more common in adults, include depression, euphoria, delusions, hallucinations, paranoia, mania, and suicidal ideation (3–5). Symptoms often start within days of treatment initiation, peak after a few weeks and resolve within 1–3 months despite ongoing treatment (3, 4). Symptom resolution has been attributed to tolerability partly due to auto-induction of efavirenz metabolism (6). However, poor long-term neurocognitive outcomes such as worsening performance in verbal fluency, working memory and speed of information processing are reported in adults after prolonged use of EFV (7). In children, some experts have predicted poor neurocognitive outcomes following long-term use (3).

The mechanism of the neuronal damage in EFV toxicity remains unknown. Disruptions in brain mitochondrial function and altered cellular bioenergetics are suggested processes. Specifically alterations in brain energy metabolism, ATP generation, reactive oxygen species (ROS) production, and creatine kinase activity have been documented (2, 8, 9). How these biochemical processes are integrated and how they impact the different cells and areas in the human brain to produce the specific symptoms is still not clear (8). In animal studies, inhibition of creatine kinase activity and mitochondrial complex IV in the cerebral cortex, striatum and hippocampus have been observed (8). These centers contain networks that regulate emotion and cognitive development. Decreased creatine kinase activity in EFV-treated mice has been associated with cognitive impairment (8). Memory deficits and depressive-like behaviors have also been noticed in rats treated with EFV. Chronic treatment of rats with EFV has produce degenerative changes in the lateral geniculate body and may be related to symptoms such as dizziness and headaches (8). In addition, EFV has lysergic acid diethylamide (LSD)-like properties which may be related to its partial agonist activity on serotonin receptors (8, 10). In rodents, the drug's interaction with these receptors appears to produce the behavioral and psychiatric profile seen with EFV usage (10).

There is wide inter-individual variation in EFV metabolism influenced by age, gender, body weight, ethnicity, concomitant diseases, and drug interactions (8, 11). The drug is mainly metabolized in the liver by the CYP450 isoenzyme CYP2B6 and to a lesser extent by CYP3A4 (12). In people of African descent, a single nucleotide polymorphism (SNP) in the CYP2B6 isoenzyme results in three different allelic variants with some individuals expressing decreased enzyme activity. These individuals are slow metabolizers of EFV and at risk of neurotoxicity (8, 13, 14).

Although this SNP is widely reported in indigenous black South Africans, (14) and cases of neuropsychiatric adverse effects in this population are published, (15–17) there is no study describing the central nervous system (CNS) complications of EFV in South African children. Thus, the scope and nature of the transient neuropsychiatric manifestations seen in children who receive EFV and how its long-term use impacts on their neurocognitive outcomes is unknown. Following the 2013 US Food and Drugs Authority approval for EFV use in children as young as 3 months old, the Southern African HIV Clinicians Society cautioned against EFV use in such young children within the sub-region, citing the pharmacogenetic variances of indigenous Africans, as well as logistic constraints for genetic testing and therapeutic drug monitoring in resource-limited settings (18).

This report describes the neuropsychiatric and neurocognitive manifestations in two South African children following treatment with EFV-based ART. In addition, we describe the acute and long-term manifestations of ten other children with suspected but not proven neurotoxicity following EFV intake.

## Method

Twelve children referred to the Neuro-HIV clinic at Red Cross War Memorial Children's Hospital (RCWMCH) in Cape Town, South Africa, were identified for this study. A detailed retrospective case report of 2 children with EFV neurotoxicity was completed. Medical records of the 10 other children with suspected neurotoxicity were reviewed. RCWMCH is the largest children's hospital in sub-Saharan Africa, providing multidisciplinary tertiary-level care within the constraints of a resource-limited country. The Neuro-HIV clinic, a referral clinic for HIV-infected children with neurological complications, reviews ~50 new cases a year, mostly diagnosed with HIV-associated neurocognitive dysfunction (HAND), HIV encephalopathy (HIVE), or other neurologic complications including epilepsy, secondary CNS infections, developmental delay, learning difficulties and behavior problems (19).

Children for the present study were identified through the clinic database and included if they were HIV-infected, treated with EFV, and experienced neuropsychiatric side effects following EFV initiation. Children were excluded if they had CNS co-infection or other chronic neurological or psychiatric illness prior to EFV initiation. Relevant data was collected including clinical information, neuroimaging, and laboratory reports. Duration on EFV-containing ART at the time of referral and plasma EFV concentrations, measured by the University of Cape Town (UCT) pharmacology laboratory, were recorded.

Neuropsychology reports, where available, were included for additional neurocognitive insight. All neuropsychology tests were conducted by a neuropsychologist. The report findings include the child's schooling history, Neuropsychological test performance and behavioral observations. The test battery included all 10 core subtests from the Wechsler Intelligent Scale for Children—Fourth UK Edition (WISC-IV) to obtain a full-scale IQ score and subscale scores in verbal comprehension, perceptual reasoning, working memory, and processing speed. Aspects of executive functioning including visuospatial planning, problem solving, frustration tolerance, and ability to understand and follow rules were assessed using the Delis-Kaplin Executive Function System Tower task (D-KEFS). Both WISC-IV and D-KEFS are internationally recognized neuropsychological tests used in multi-cultural and lingual populations and have been previously used in South African samples (20–22). In children whose first language is not English, an interpreter was used during the testing. Developmental assessment reports were included for children assessed by the child development service using the Molteno Adapted Scale, a locally developed screening measure with good test accuracy and acceptable sensitivity, specificity, and predictive values for developmental delay on the Griffiths Mental Development Scales (23). School reports were included where available.

All caregivers gave verbal consent for the inclusion of the children's details in the clinic database. The UCT Faculty of Health Sciences Human Research and Ethics Committee approved the study (HREC 479/2016) and waived the need for written informed consent. The RCWMCH Research Committee granted permission for the study.

## Results

Between 2008 and 2014, 12 children (7 females) were referred with suspected EFV neurotoxicity. The children were aged between 3 and 12 years (median 8.4). Eight children were of indigenous African (black) ancestry and 4 of mixed ancestry.

Acute neuropsychiatric manifestations were reported in 6 children; namely drowsiness (5/12), seizures (4/12), sleep disturbances (2/12), personality changes (2/12), acute ataxia (1/12), and slurred speech (1/12). All 6 children had multiple complaints: four had 2 manifestations, one had 3 and another had 4. These manifestations occurred within 2–8 (mean 5) weeks after starting EFV.

Eleven children had neuropsychology, developmental, or academic assessments −6 children had neuropsychology assessments, 4 developmental evaluations and 7 school reports. These assessments were used to determine frequencies of long-term neurocognitive manifestations which were noted after 6–72 (mean 31) months on EFV and included poor performances in processing speed (5/11), intelligent quotient (IQ) (5/11), attention span (4/11), working memory (4/11), verbal comprehension (3/11), perceptual reasoning (2/11), executive functioning (1/11), and visuospatial ability (1/11) as well as delayed fine motor skills (3/11) and speech/language development (3/11); leading to academic underachievement in 6 children. Multiple complaints were common: one child had 6, four had 5 and two had 3 manifestations. Of the 7 children whose school reports were available, 6 were not coping with mainstream curriculum, with 5 repeating various grades.

Eight children had neuroimaging studies of whom 3 had brain CT scans (all normal) and 5 had brain MRI (3 normal, 1 had non-specific signal changes, and 1 had old infarcts).

The total duration on EFV at the time of referral to neurology was known for 10 children (range 6–72 months, mean 31 months) and unknown for 2 children but treatment was assumed to be established as neither reported acute manifestations.

Mid-dose plasma EFV concentration was done for 10 children. Levels ranged from 0.8 to 69 mg/L (normal reference range 1–4 mg/L). Two children had toxic plasma EFV concentrations (69.0 and >20.0 mg/L, respectively), seven had levels within the normal reference range while one had sub-therapeutic level.

Illustrative case summaries for the 2 children with toxic plasma EFV levels are presented below, while Table 1 provides overviews of all 12 children.

**Table 1:** Demography, relevant history, and findings.

**No**	**Age, Race, and Year presented**	**Reason(s) for referral to neurology**	**Socioeconomic status and relevant history**	**Clinical, Laboratory, and Neuro-radiological findings**	**School report, Developmental assessment, and Neuropsychology assessment (age at report)**	**Total duration on EFV**	**Plasma EFV level (mg/L)**	**Treatment outcomes**
1	3–4 years, African, 2008	• Drowsiness • Seizures • Ataxia • Slurred speech (few weeks after starting EFV)	• Lived with mother (HIV+) and sister (HIV-). • Father died (HIV-related) when he was 3–4 years old. • Diagnosed HIV+ at age <1 year • Started ART at age 1–2 years.	**Clinical:** Underweight, WHO stage 3 disease. **Lab:** CD4 count 310 cells/μL (11%) at ART initiation and 1,549 cells/μL (37%) at referral. VL <50 copies/mL at referral. **EEG:** non-specific generalized slowing. **Audiology:** normal hearing **Neuroimaging:** normal brain CT scan	**School report (grade 2, age 8–9 years)**: Functioning below average in all areas. Had poor numeracy skills. **Neuropsychology report (age 9–10 years):** • borderline to extremely low VC • low average to extremely low PR • extremely low WM • borderline to extremely low PS • low achievement on EF • full IQ score 48 (extremely low).	20 mo	69.00	• Diagnosed as EFV neuro-toxicity. • Discontinued EFV treatment. EFV levels decline over time. • Drowsiness, ataxia and slurred speech resolved. • Seizures treated with valproate
2	3–4 years, African, 2008	• Insomnia (2 mo after starting EFV) • Seizures (GTCS) • Impaired concentration • (6 mo after starting EFV)	• Lived with mother (HIV+). • Father was not involved with care. • No antenatal PMTCT but had AZT after birth for 6 weeks. No NVP. • Diagnosed HIV+ at age <1 year. • Started ART at age 1–2 year. • History of poor adherence to ART.	**Clinical:** Well-grown, hyperactive child with poor concentration. WHO stage 2 disease. **Lab:** CD4 count 510 cells/μL (11.3%) at ART initiation. **EEG:** Normal awake and sleep EEG. **Neuroimaging:** Normal brain CT scan	Not available	22 mo	Not done	• Diagnosed as HAND. • Continued EFV treatment. • Sleep improved over time • Seizures treated with valproate.
3	7–8 years, African, 2009	• Seizures (staring and GTCS) • Impaired concentration • (6 weeks after starting EFV)	• Mother deceased (HIV-related). • Father was not involved initially, but later came back into picture when he was 7–8 years • Placed in institutional care at age 3–4 years and moved in with father at age 7–8 years. • Diagnosed HIV+ at age <1 year. • Started ART at age 6–7 years.	**Clinical:** Microcephalic, ADHD, mild to moderate speech delay. **Lab:** CD4 count 321 cells/μL (14.1%) at ART initiation and 1,099 cells/μL (33.6%) at referral. **EEG:** Normal awake and sleep EEG. **Audiology:** normal hearing **Neuroimaging:** Calcified mastoid air cells on CT scan, normal brain parenchymal.	**Developmental assessment (age 7–8 years):** Delayed fine motor (64 mo at chronologic age of 86 mo) and speech development (66 mo at chronological age of 86 mo). **Conner's questionnaire:** completed by both teacher and carer (at age 6y 3mo) suggestive of ADHD.	9 mo	2.70	• Diagnosed as HIVE. • Continued EFV treatment • Seizures treated with valproate • ADHD treated with methylphenidate • Had speech therapy.
4	10–11 years, African, 2010	Poor concentration affecting school work	• Forster care. Mother deceased, no information on father. • No details on when and how HIV was diagnosed HIV+ and duration on ART.	**Clinical:** Stunted, long tract signs in lower limbs. **Lab:** CD4 count or VL details not available **Audiology:** Normal audiogram (had poor concentration during testing **Neuroimaging:** Not done	**School report (grade 2, age 10–11 years):** Struggling with mainstream curriculum (repeating grade 2)	Not known	1.04	• Diagnosed as HIVE/HAND. • Continued EFV treatment
5	6–7 years Mixed ancestry 2012	• Poor concentration • ADHD	• Forster care. Both parents deceased (HIV-related). • History of alcohol exposure *in utero*. • Diagnosed HIV+ at age 1–2 years • Started ART at age 2–3 years	**Clinical:** Stunted, microcephalic, FAS facies, ADHD, and long tract signs. **Lab:** CD4 count 749 cells/μL (17.1%) at ART initiation. **Audiology:** Normal hearing but slow in responses (needed reinforcement during testing). **Neuroimaging:** normal brain MRI	**Neuropsychology report (age 7–8 years):** • Borderline VC • Borderline PR • Low WM • Extremely low PS • Verbal IQ 70 (borderline) • Performance IQ 70 (borderline) • Full scale IQ 62(extremely low) **Conner's questionnaire:** completed by foster parent and teacher (at age 6–7 years) suggest ADHD	Not known	2.04	• Diagnosed as HIVE, FAS, ADHD. • Continued EFV treatment. • ADHD treated with methylphenidate. • Special needs school recommended
6	8–9 years, African, 2012	• Drowsiness • Difficulty arousing from sleep • (few weeks after starting EFV) • Poor concentration • Poor memory affecting school work	• Forster care. Both parents deceased (HIV-related) • History of prenatal alcohol exposure. • Diagnosed HIV+ at age 3–4 years • Started ART at age 6–7 years.	**Clinical:** Stunted and underweight. No features of FAS. No focal signs. Noted to be drowsy during clinic visit. **Lab:** CD4 percentage of 34% at time of referral. **Audiology:** Normal testing. **Neuroimaging:** Non-specific T2W high signal abnormality in the right peritrigonal white matter on brain MRI.	**School report (grade 2, age 8–9 years):** Not coping with mainstream curriculum. Repeated grade 2. **Neuropsychology report (age 8 years 9 mo):** • low AS, WM and PS • borderline full-scale IQ (70) • Was drowsy during testing.	28 mo	>20.00	• Diagnosed as EFV neuro-toxicity. • Discontinued EFV treatment. EFV levels declined over time. • Special needs school recommended
7	10–11 years, African, 2013	• Poor concentration • Poor memory affecting school work.	• Lived with parents, (both HIV+), and 2 siblings (both HIV-). • Had generally good health until age 8–9 years when she was diagnosed HIV+. • Started ART at 8–9 years.	**Clinical:** Well grown. Had low central tone, brisk reflexes with spread, and crossed adductors. Mirror movement with finger apposition. **Lab:** CD4 count 158 cells/μL at ART initiation. **Audiology:** Normal testing. **Neuroimaging:** Brain MRI showed old lacuna infarct	**School report (grade 2, age 10–11 years)**: Difficulties with English language and Mathematics (repeated grade 2). **Developmental assessment (age 10–11 years)**: Poor fine motor skills. **Neuropsychology report (age 10 years 7 mo):** • Extremely low AS, PS, VC, PR, verbal IQ (55), performance IQ (69) and full-scale IQ (60) • intact memory	24 mo	1.92	• Diagnosed as HAND. • Continued EFV treatment.
8	11–12 years, Mixed ancestry, 2013	• Poor concentration • Poor school performance	• Lived with parents (both HIV+) and sibling (HIV–). • Diagnosed HIV+ at age 4–5 years. • Started ART at 5–6 years.	**Clinical:** Well grown. Had long tract signs in all limbs. **Lab:** CD4 count 202 cells/μL (7.9%) at ART initiation and 1,602 cells/μL (38%) at referral **Audiology:** Normal testing. **Neuroimaging:** Normal brain MRI	**School report (grade 3, age 12–13 years):** Struggling with mainstream curriculum (repeated grades 1 and 3)	64 mo	3.90	• Diagnosed as HAND/HIVE. • Continued EFV treatment.
9	5–6 years, African, 2013	• Withdrawn behavior • Emotional outburst (notice within 1year after EFV)	• Failed PMTCT. • Separated from biological mother on day 2 of life and placed in “place of safety” until adopted at age 4 mo. • Normal early development. • Diagnosed HIV+ age <1 year • Started on ART at age 2–3 years.	**Clinical:** Well-grown, normal neurological examination. **Lab:** CD4 count or VL details not available **EEG:** Normal background, sharply contoured activity noted bilaterally. **Neuroimaging**: not done	**Developmental assessment (age 5–6 years)**: Development assessed to be age appropriate. Draw-a-person score of 67 mo (at chronologic age of 65 mo old)	24 mo	1.28	• Diagnosed as attachment disorder and referred to psychiatry. • Continued EFV treatment. • Had gradual improvement in behavior.
10	8–9 years, Mixed ancestry, 2014	• Poor concentration • Emotional outburst • Personality changes (noticed 2mo after EFV)	• Lived with mother (HIV+) and 2 siblings (both HIV-). • Father was not involved. • Diagnosed HIV+ at age 6–7 years. • Started ART at age 8–9 years.	**Clinical:** Microcephalic. Had long tract signs. **Lab:** CD4 count 1003 cells/μL (29%) at time of referral **Audiology:** normal hearing **Neuroimaging**: Normal brain MRI	**Neuropsychology report (age 8–9 years):** • Low average general intellectual functioning • Low PS • severe to profound impairment in AS and visuospatial ability • borderline WM, VM and EF	6 mo	Not done	• Diagnosed as HIVE. • Continued EFV treatment
11	9–10 years African 2014	Poor concentration leading to school failure	• Lived with mother (HIV+). • Father deceased (HIV status unknown). • Started ART at age 1–2 years. • Developed hypersensitivity (rash) to ABC and lipoatrophy on AZT. At time of referral, was on 3TC/EFV.	**Clinical:** Well-grown. Normal neurological examination. **Lab:** CD4 count 1260 cells/μL (26.7%), VL = 1,400,000 copies/mL at ART initiation. **Audiology**: normal hearing. **Neuroimaging:** Not done.	**Developmental assessment (age 3–4 years):** At chronologic age of 45 mo, had significant delay in speech (+/– 30 mo) and fine motor development (30–36 mo). **School report (grade 3, age 9–10 years):** • repeated grade 1 • was struggling with mainstream curriculum in grade 3. **Neuropsychology report** **(age 9–10 years):** • Marked difficulty with AS, PS and language • Memory (verbal and visuospatial) spared.	72 mo	0.80	• Diagnosed as HAND. • Continued EFV treatment. • Had speech therapy. • Special needs school recommended.
12	10–11 years, Mixed ancestry, 2014	• Seizures (staring) • Drowsiness few weeks after starting EFV	• Lived with grandparents. • Mother (HIV+) was on illicit drugs (died when patient was 6–7 years old). • Father was not involved. • Diagnosed HIV+ at age 1–2 years • Started on ART at age 1–2 years • History of poor adherence to ART.	**Clinical:** Microcephalic with long tract signs. WHO stage 4 disease. **Lab:** CD4 count 662 cells/μL (5.45%) at ART initiation **EEG:** Awake EEG normal **Neuroimaging:** Not done	**School report (grade 4, age 10–11 years):** Pursuing mainstream curriculum with average performance.	26 mo	1.70	• Diagnosed as HIVE. • Continued EFV treatment • Seizures treated with valproate

***Key:** 3TC, lamivudine; ABC, abacavir; ADHD, attention deficit hyperactivity disorder; ART, antiretroviral therapy; AZT, zidovudine; EEG, electroencephalogram; EFV, efavirenz; FAS, fetal alcohol syndrome; GTCS, generalize tonic-clonic seizures; HAND, HIV-associated neurocognitive dysfunction; HIV+, HIV-positive; HIV-, HIV-negative; HIVE, HIV encephalopathy; mo, month(s); NVP, nevirapine; PMTCT. Prevention of mother to child transmission; VL, viral load; y, year(s)*.

***Key for neuropsychology reports:** AS, attention span; EF, executive functioning; IQ, intelligence quotient; PR, perceptual reasoning; PS, processing speed; VC, verbal comprehension; WM, working memory*.

**Patient 1** is a child of indigenous African ancestry referred in 2008 at 3–4 years of age. He was diagnosed with pulmonary tuberculosis and HIV infection in infancy. He commenced an ART regimen of stavudine (d4T), lamivudine (3TC) and EFV at 1–2 years of age. Both his parents were HIV-infected, and his father died of HIV-related complications 2 years later.

One month after starting ART, he had his first generalized tonic-clonic seizure (GTCS) during a febrile illness. At 2–3 years of age, he experienced multiple afebrile GTCS and drowsiness. He was ataxic, had slurred speech, and regression of his motor milestones. His mother reported that the drowsiness was present since he started ART, but the ataxia and slurred speech were new. His HIV viral load was <50 copies/mL and his CD4 count was 1549 cells/μL (37%). His cerebrospinal fluid (CSF) had no cells, normal chemistry and was negative for herpes simplex viruses 1 & 2, John Cunningham virus and *Cryptococcus neoformans*. His brain CT scan was normal. His EEG showed non-specific generalized slowing and no evidence of non-convulsive status epilepticus (NCSE). Antiepileptic medication was not initiated. The ataxia resolved over a period of 1 month.

He was re-admitted to hospital 7 months later with acute ataxia and drowsiness. His mother had misunderstood his medication instructions and had given him a daily EFV dose of 650 mg instead of the prescribed 250 mg during the 2-week period prior to admission. His CSF analysis and repeat brain CT scan were normal. A repeat EEG was normal. His plasma EFV level was 69.0 mg/L and efavirenz was discontinued. Over the ensuing 2-week period his plasma EFV level declined to 15.2 mg/L, and the ataxia resolved.

Efavirenz was restarted after 3 weeks. Repeat plasma EFV levels performed at 1 and 3 weeks remained elevated at 12.9 and 12.0 mg/L, respectively. However, treatment with EFV was continued. Two months later, he was re-admitted with prolonged GTCS requiring phenobarbital loading. After recovery, he was ataxic. EEG showed generalized slowing and no evidence of NCSE. Efavirenz levels were not performed during this admission. He commenced sodium valproate for seizure management.

Four months later, he was re-admitted with GTCS and ataxia. Plasma EFV level was 36.4 mg/L. He was finally diagnosed as EFV neurotoxicity, efavirenz was permanently discontinued, and replaced with lopinavir/ritonavir (LPV/r). Repeated plasma EFV level 5 days after discontinuation was 28.0 mg/L. Two months after stopping EFV there were no further seizures, drowsiness or ataxia, and the plasma EFV level was 0.4 mg/L. He was thought to be a slow metabolizer.

Over the next 2 years, he remained virologically suppressed on d4T/3TC/LPV/r. His mother who herself continued an EFV-containing ART regimen had an elevated mid-dose plasma EFV level of 4.3 mg/L recorded during one of her clinic visits.

At the Neuro-HIV clinic, his dosage of sodium valproate was optimized, and he had no further seizures, drowsiness, or ataxia. An audiogram showed normal hearing. Sodium valproate was stopped at age 7 years. A year later, he repeated grade 1 with a school report indicating that he was functioning below average in all areas and had poor numeracy skills. Neuropsychology assessment performed at age 9 years documented a full-scale IQ score in the extremely low range with extremely low verbal comprehension, perceptual reasoning, working memory and processing speed, and poor executive functioning.

**Patient 6** is an African female aged 8–9 years when referred in 2012. She was in foster care as both parents were deceased from HIV-related complications. She had prenatal exposure to alcohol and was diagnosed with pulmonary tuberculosis and HIV infection at age 3–4 years old.

At 6–7 years of age she commenced first-line ART (d4T/3TC/EFV). After 21 months of treatment, d4T was changed to abacavir (ABC) due to development of lipodystrophy. After 28 months on EFV she was referred to the Neuro-HIV clinic because of poor memory, drowsiness during school hours and nocturnal enuresis. Despite sleeping adequately at night, it was difficult to arouse her in the morning and she often fell asleep in school. Drowsiness and sleep disturbances manifested within a few weeks after starting first-line ART. She struggled academically in second grade mainstream education, which her teacher attributed to poor concentration and memory. Although she had no formal cognitive assessment prior to EFV initiation, both foster parent and school teacher noticed changes in her concentration and memory.

At presentation to the Neuro-HIV service, aged 8–9 years, she was stunted, underweight for age, and microcephalic. She had no features of fetal alcohol syndrome and no focal neurological deficits. Her “draw-a-person score” was 5 years. Her CD4 percentage was 34%, within normal limits.

Her mid-dose plasma EFV concentration, performed 2 weeks prior to referral, was >20.0 mg/L. EFV neurotoxicity was diagnosed and EFV discontinued. Repeat plasma EFV level 2 weeks after discontinuation was 0.74 mg/L. Brain MRI showed non-specific T2-weighted signal abnormalities in the right peritrigonal white matter. Neuropsychology assessment at age 8.8 years documented poor performances in attention, working memory and processing speed. Her overall IQ was in the borderline range. Five weeks after stopping EFV, she was no longer sleepy during the daytime. She continued treatment with ABC/3TC/LPV/r and recommendation was made for placement in special school.

## Discussion

The children in this study all had complex socio-economic circumstances such as orphanhood, having uninvolved parents, or being in foster care. Prenatal alcohol exposure was reported in one child. These factors together with the neuroinflammatory effect of HIV itself, and the secondary neurological complications result in a complex interplay which impacts on the behavior of the affected children. Frontal lobe dysexecutive syndrome in HIV-infected children and adolescents may result from white matter damage leading to cognitive and behavioral challenges, including poor concentration and hyperactivity (24) and could contribute to the neuropsychiatric manifestations seen in this cohort. However, most of the acute manifestations in this study commenced within a few weeks of EFV initiation.

Some of the children described in this report had abnormal neurological examinations, significant neurodevelopmental delay, microcephaly, stunting, or major learning disorders and were diagnosed with HIV encephalopathy (HIVE). These features are probably attributable to delayed initiation of ART in children with perinatal HIV infection as evidenced by the low CD4 counts or high viral loads prior to ART initiation, and possibly worsened by exposure to EFV. In contemporary clinical practice with improved global healthcare and access to ART, and early ART initiation, the natural course of the disease has changed, and the incidence of HIVE is on the decline (25).

The 2 children who had high plasma EFV levels were both indigenous Africans. Their clinical phenotypes were consistent with slow metabolizers although CYP2B6 genotyping was not performed. They both developed neuropsychiatric manifestations within a few weeks of EFV initiation that did not resolve until EFV was discontinued. Though there were no neuropsychology assessments prior to EFV initiation, both children performed poorly in all the domains when assessed later.

Ten other children were referred to the neurology service with suspected EFV neurotoxicity. Although they had normal or subtherapeutic EFV levels, their neuropsychiatric manifestations were noticed after EFV initiation. A presumptive diagnosis of EFV neurotoxicity could have been reached if EFV treatment was stopped and later re-introduced to observe for symptom resolution after stopping and recurrence after re-starting treatment with EFV. Unfortunately, these evaluations were not performed as ART withdrawal without replacement would have been considered as poor continuity with its associated poor outcomes. These children were later diagnosed as either HAND, HIVE, or behavioral disorders and continued treatment with EFV. Their acute manifestations resolved over time despite continuous treatment with EFV, as well as the other specific manifestations such as seizures and ADHD which were treated with appropriate medications.

Among the suspected acute neuropsychiatric manifestations seen, drowsiness, and impaired concentration were most common, occurring in 5 children, including the two with high EFV concentrations. Drowsiness is reported as the most common acute CNS manifestation of EFV accounting for up to 50% of cases (5, 26). It typically resolves spontaneously within 1–2 months due to tolerability and does not lead to EFV discontinuation (3, 4). It may however be missed or not be reported, especially in children. Night-time EFV dosing reduces the effect on patient concentration (3).

Seizures were the second most common manifestation in our cohort, reported in 4 children. All were treated with valproate with good outcomes, although 1 child required EFV discontinuation in addition to valproate. The incidence of seizures is generally higher in HIV-infected children (27). Our study numbers were too small to establish a causal relationship with EFV initiation and seizures. However, the published literature documents 3 cases of seizures in South African children following EFV initiation that required treatment discontinuation before seizure control was achieved (15, 17). All 3 children expressed the CYP2B6 polymorphism rendering them slow metabolizers. Status epilepticus was reported in an adult following marked EFV toxicity that also required treatment discontinuation (28). In other instances children continued EFV treatment as seizures were controlled (29).

Sleep disturbances are commonly reported and were documented in 2 of our children (30). In one child, this resolved despite continued EFV administration, while in the other it persisted until EFV was discontinued. Sleep disturbances may not be transient for slow metabolizers who maintain high and toxic plasma levels even after ingestion of therapeutic doses. There were no nightmares or vivid dreams reported by our children, but caregivers may not have been asked about their presence. These are commonly reported by adults or adolescents (30, 31).

Ataxia was noticed in one patient with high plasma EFV concentration. It improved gradually with corresponding decline in the plasma EFV levels after treatment discontinuation. Ataxia has been reported in other South African children with supra-therapeutic efavirenz levels (16).

Personality changes such as emotional outburst and withdrawn behavior were seen in 2 children who both continued on EFV with symptom resolution over time. However, psychiatric reactions such as hallucinations, depression, and suicidal ideation were not reported by our children, but are recognized in adolescents and adults (32, 33).

Poor neurocognitive outcomes following long-term EFV use, particularly decreased verbal fluency, working memory, and processing speed, are reported in adults (7). In our cohort, we recorded poor performances in all cognitive domains and in academic records. Developmental assessments also revealed delayed fine motor, speech and language development in 3 children. In children, such outcomes are often attributed to HAND or HIVE (34, 35). While these developmental challenges cannot solely be attributed to EFV as children with HIVE typically have developmental delay (19, 35), EFV may have exacerbated these outcomes.

## Limitations of the Study

The content of this case series is limited by the retrospective data collection. There were no cognitive or developmental assessments documented at the time of EFV initiation. Further, only 6 and 4 children, respectively had these assessments as part of their evaluation after referral. There was no controlled timing for the plasma EFV levels, and the study design did not include a control group of EFV-naïve children. It is therefore not possible to establish a causal relationship between efavirenz neurotoxicity and neuropsychiatric manifestations. Furthermore, CYP2B6 genotyping could not be performed in our setting and would have been of great interest especially in the 2 children who had toxic plasma EFV levels.

## Conclusion

In this retrospective study of 12 HIV-infected children with complex socio-economic status and on EFV-based ART, a wide spectrum of neurological and neurocognitive manifestations were documented, some precipitated by EFV exposure and others appear to have been aggravated by EFV exposure. Two children with toxic EFV levels experienced neuropsychiatric manifestations related to EFV exposure. An adverse EFV response was suspected for the remaining children. These manifestations could be from the complex interplay of the difficult socio-economic circumstances, the neuroinflammatory effect of HIV itself, the secondary complications from opportunistic infections and other treatments besides EFV. Prospective controlled studies are needed to further explore the relation between neurological, neuropsychiatric and long-term neurocognitive manifestations in HIV-infected children, and EFV exposure. Pharmacogenetic analysis is important to understand the role and contribution of genetic polymorphism in future studies.

## Data Availability

All datasets generated for this study are included in the manuscript and/or the Supplementary Files.

## Ethics Statement

This study was carried out in accordance with the declaration of Helsinki. All caregivers gave informed consent for the inclusion of their children's details in the clinic database. The UCT Faculty of Health Sciences Human Research and Ethics Committee approved the study (HREC 479/2016) while the Red Cross War Memorial Children's Hospital Research Committee granted permission for the study.

## Author Contributions

CH wrote the original draft, completed the literature search, collected the patient data, and summarized the cases. BE provided advice on HIV treatment aspects of the manuscript, critiqued the content, and proofread the manuscript. NI provided critical advice relating to the neuropsychiatric assessments and contributed to the manuscript. JW conceptualized the study, supervised the data collection, contributed to the manuscript content, and approved the final version.

### Conflict of Interest Statement

The authors declare that the research was conducted in the absence of any commercial or financial relationships that could be construed as a potential conflict of interest.

## Abbreviations

3TC: lamivudine
ABC: abacavir
ART: anti-retroviral therapy
d4T: stavudine
EEG: electroencephalogram
EFV: efavirenz
HAND: HIV-associated neurocognitive dysfunction
HIVE: HIV encephalopathy
LPV/r: lopinavir/ritonavir
SNP: single nucleotide polymorphism.
